# Reducing asthma attacks in disadvantaged school children with asthma: study protocol for a type 2 hybrid implementation-effectiveness trial (Better Asthma Control for Kids, BACK)

**DOI:** 10.1186/s13012-024-01387-3

**Published:** 2024-08-15

**Authors:** Amy G. Huebschmann, Nicole M. Wagner, Melanie Gleason, John T. Brinton, Michaela Brtnikova, Sarah E. Brewer, Anowara Begum, Rachel Armstrong, Lisa Ross DeCamp, Arthur McFarlane, Heather DeKeyser, Holly Coleman, Monica J. Federico, Stanley J. Szefler, Lisa C. Cicutto

**Affiliations:** 1 Anschutz Medical Campus Department of Medicine, Division of General Internal Medicine, University of Colorado, 12631 E. 17th Ave., Mailstop B180, Aurora, CO USA; 2 Adult and Child Center for Outcomes Research and Delivery Science (ACCORDS), 1890 Revere Ct, Suite P32-3200, Mailstop F443, Aurora, CO 80045 USA; 3 Ludeman Family Center for Women’s Health Research, Aurora, CO USA; 4 Department of Pediatrics, University of Colorado School of Medicine, Aurora, USA CO; 5 Department of Family Medicine, University of Colorado School of Medicine, Aurora, CO USA; 6 National Jewish Health and University of Colorado College of Nursing and Clinical Sciences, Aurora, CO USA; 7 Breathing Institute, Children’s Hospital Colorado, 13123 East 16Th Avenue, Mailstop B395, Aurora, CO 80045 USA; 8 Trailhead Institute, 1999 Broadway Suite 200, Denver, CO 80202 USA

**Keywords:** Asthma, Social determinants of health, Implementation science, School health services, Health equity, Child health

## Abstract

**Background:**

Asthma is a leading cause of children’s hospitalizations, emergency department visits, and missed school days. Our school-based asthma intervention has reduced asthma exacerbations for children experiencing health disparities in the Denver Metropolitan Area, due partly to addressing care coordination for asthma and social determinants of health (SDOH), such as access to healthcare and medications. Limited dissemination of school-based asthma programs has occurred in other metropolitan and rural areas of Colorado. We formed and engaged community advisory boards in socioeconomically diverse regions of Colorado to develop two implementation strategy packages for delivering our school-based asthma intervention — now termed “Better Asthma Control for Kids (BACK)" — with tailoring to regional priorities, needs and resources.

**Methods:**

In this proposed type 2 hybrid implementation-effectiveness trial, where the primary goal is equitable reach to families to reduce asthma disparities, we will compare two different packages of implementation strategies to deliver BACK across four Colorado regions. The two implementation packages to be compared are: 1) standard set of implementation strategies including Tailor and Adapt to context, Facilitation and Training termed, BACK-Standard (BACK-S); 2) BACK-S plus an enhanced implementation strategy, that incorporates network weaving with community partners and consumer engagement with school families, termed BACK-Enhanced (BACK-E). Our evaluation will be guided by the Reach, Effectiveness, Adoption, Implementation, and Maintenance (RE-AIM) framework, including its Pragmatic Robust Implementation Sustainability Model (PRISM) determinants of implementation outcomes. Our central hypothesis is that our BACK-E implementation strategy will have significantly greater reach to eligible children/families than BACK-S (primary outcome) and that both BACK-E and BACK-S groups will have significantly reduced asthma exacerbation rates (“attacks”) and improved asthma control as compared to usual care.

**Discussion:**

We expect both the BACK-S and BACK-E strategy packages will accelerate dissemination of our BACK program across the state – the comparative impact of BACK-S vs. BACK-E on reach and other RE-AIM outcomes may inform strategy selection for scaling BACK and other effective school-based programs to address chronic illness disparities.

**Trial registration:**

Clinicaltrials.gov identifier: NCT06003569, registered on August 22, 2023, https://classic.clinicaltrials.gov/ct2/show/NCT06003569.

**Supplementary Information:**

The online version contains supplementary material available at 10.1186/s13012-024-01387-3.

Contribution to the literature:

- In prior work, we developed, refined and implemented the Better Asthma Control for Kids (BACK) program in urban Colorado school districts where it decreased asthma exacerbations and has been sustained through support from staff in local school districts and state public health department grants.
- In four geopolitically diverse (urban and rural) areas, this project will test the comparative impact of implementing the evidence-based BACK program to reduce asthma disparities with two different packages of implementation strategies.
- Data from this trial will inform a “dissemination playbook” to accelerate future dissemination of BACK to communities experiencing pediatric asthma care inequities.

## Background

Asthma disproportionately affects children living in historically marginalized and under-resourced communities [1]. Disparities in asthma outcomes include higher mortality rates, worse asthma control, greater likelihood of emergency visits, and higher rates of school absenteeism [2–4]. Pediatric asthma disparities are partly driven by unmet Social Determinants of Health (SDOH) needs, such as lack of insurance and transportation that lead to fewer preventive care visits and more emergency visits and hospitalizations [1, 5–11]. In addition to health impacts, poor asthma control causes educational disparities through missed school days, increased fatigue, and difficulty concentrating due to interrupted sleep that negatively impact school performance [12–15]. As a result, asthma is one of seven educationally relevant health disparities that school leaders prioritize [16, 17].

**Fig. 1:**
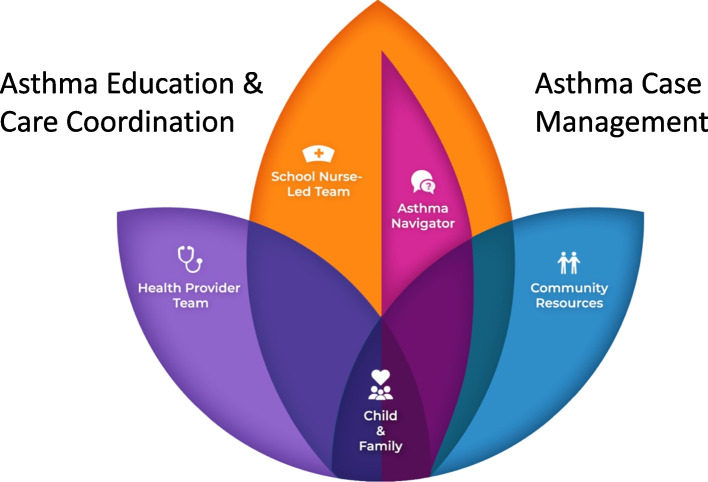
Key roles in our patient-centered BACK program. Our BACK program puts the child and family at the center of everything we do. The role of the ANav is to support the school nurse-led team to deliver the core functions of BACK. This includes the ANav assisting the school nurse-led team with asthma education for the child/family, and asthma case management and care coordination between the child/family, health provider team, and community resources for SDOH. The ANav links families to community resources for SDOH to address financial constraints of asthma care, such as inadequate insurance coverage, lack of affordable transportation, and difficulty affording medications

Over the past 18 years, we have developed an effective school-based asthma program that reduced asthma exacerbations and school absences [18–21]. These positive outcomes have been achieved by identifying eligible children with poor asthma control through routine school registration processes, and by a community Asthma Navigator (ANav) providing asthma care coordination and case management (see Table 1). Our approach includes coordination across families, schools, health care providers, and community agencies with resources to address unmet SDOH needs – the latter is key to address disparity drivers such as inadequate access to healthcare and difficulty affording medications (Fig. 1) [20, 22–24]. Care coordination with health care provider teams is critical to ensure that the necessary asthma care plan and medications are available at school for students, and allows school nurses to alert providers to asthma care needs or gaps. These core functions of our school-based Better Asthma Control for Kids (BACK) program have been identified as effective in systematic reviews and other studies of school-based asthma management programs [25–34].

**Table 1:** BACK Core Functions, Outcomes and Comparison to the Literature

**Core Functions of BACK intervention**	**Outcomes from our previous work**	**Literature supporting core functions** [4, 25–27]
**BACK functions of Asthma case management, care coordination, and education:** • School nurse (RN) functions: ◦ Identify children with asthma ◦ As capacity allows, identify children with poorly controlled asthma who are eligible for BACK • ANav functions are to support the school RN-led team: ◦ Assess asthma control to confirm student eligibility for BACK ◦ Ensure completed asthma care plan and rescue inhaler are at school ◦ Tailored instruction to develop the student’s and caregiver’s asthma knowledge and self-management skills ◦ Provide asthma case management and care coordination across family, school, and health care providers ◦ Standardized SDOH assessment and management	**Outcomes of delivery of core functions by RN and ANav** • ↓Asthma hospitalizations [20] • ↓Asthma urgent care or Emergency Department (ED) visits [20] • ↓Oral steroid bursts • ↑Asthma Control [18] • ↑Asthma knowledge [19, 20] • ↑Inhaler technique • ↑Self-management skills • ↑Quality of life • ↓School absenteeism [18, 20] • ↓Caregiver work loss	**Evidence for effectiveness of asthma self-management components consistent with BACK** • ↓Asthma hospitalizations [25, 26] • ↓Acute asthma exacerbations – including urgent care/emergency visits [25, 26] • ↑Child activity level [25, 26] • ↑Asthma knowledge [25, 26] **Randomized controlled trial (RCT) evidence for effectiveness of in-person health navigation SDOH screening on social need and child health** • ↓Caregiver reported social needs [35] • ↑Child’s overall health status (per caregiver report) [35]

A challenge for sustained implementation is that BACK requires ongoing public health funding in most of the school districts where it has been implemented; thus, BACK could benefit from additional community-engaged efforts to support sustainability. In addition, relatively little implementation of school-based asthma programs has occurred in rural and smaller urban areas, and current implementation guides cannot differentiate the potential benefit and cost for smaller versus larger school districts [36]. Thus, the key next steps to scale BACK more broadly are to test the relative impact and cost of alternate implementation strategy packages for delivering BACK, and to develop a more robust implementation guide that allows future adopters to consider the tradeoffs of cost and impact of alternate implementation approaches.

To take these next steps, we leveraged funding from the Disparities Elimination through Coordinated Interventions to Prevent and Control Heart and Lung Disease Risk (DECIPHeR) Award to conduct a 3-year community-engaged planning phase in regions across Colorado where we had not previously implemented BACK [37, 38]. Specifically, we completed an Exploration phase [38] and Preparation phase [38] guided by the implementation determinants in the Pragmatic Robust Implementation Sustainability Model (PRISM) [39]; the activities included**:** 1) regular meetings with multi-sectoral community advisory boards (CABs) representing community, family, health care and school partners, 2) conduct of needs assessments to identify local needs, priorities and resources, and 3) tailoring BACK implementation strategies to local context with input from our CABs. Based on CAB input, we added a key non-profit partner (Trailhead Institute©) to our implementation team to build organizational capacity to connect with local public health and community organizations across the state, as part of our implementation and sustainment efforts.

In this NIH-funded hybrid type 2 implementation-effectiveness trial, we will implement BACK in four diverse regions of Colorado in school districts that have high rates of unmet SDOH needs, based either on free-reduced lunch rates or rural status [40]. Students with poorly controlled asthma [41] will be eligible to enroll in the study. We will evaluate the impact of implementing BACK with two different implementation packages: either 1) a standard set of implementation strategies including Tailor and adapt to context, Facilitation and Training, termed BACK-Standard (BACK-S), or 2) BACK-S strategies plus enhanced implementation strategies, incorporating two of the Expert Recommendations for Implementing Change (ERIC) strategies [42] of “network weaving” and “consumer engagement”, termed BACK-Enhanced (BACK-E).

## Methods

### Study aims and hypotheses

**Fig. 2:**
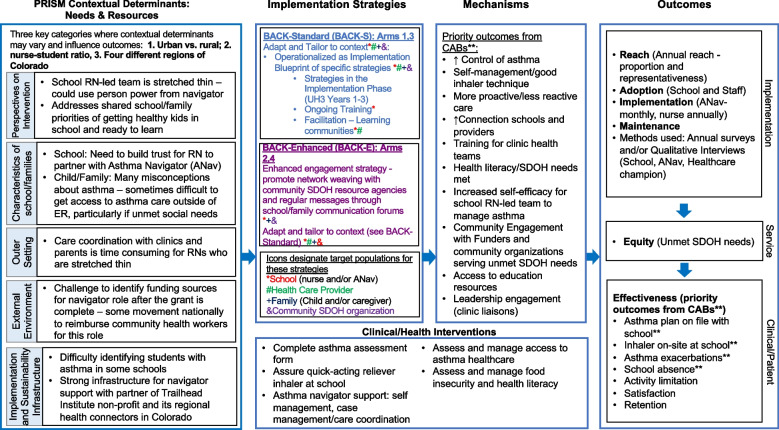
Implementation Research Logic Model. Abbreviations – PRISM (Pragmatic Robust Implementation Sustainability Model), RN (School Nurse), ANav (Asthma Navigator), BACK (Better Asthma Control for Kids), SDOH (Social Determinants of Health), CAB (Community Advisory Board)

Our primary implementation aim is to compare the reach to students with uncontrolled asthma between BACK-E and BACK-S. We hypothesize that student reach will be significantly greater when delivered using BACK-E as compared to BACK-S. Our secondary aim is to determine and compare annual asthma exacerbation rates (i.e., exacerbations/year) in students randomized to either study arm (i.e., effectiveness). We hypothesize that BACK, delivered either as BACK-E or BACK-S, will be more effective than usual care at reducing annual asthma exacerbation rates. Our third aim will identify PRISM contextual factors [39, 43] (see Fig. 2) that predict student reach and retention, school-level adoption, costs to future adopters (schools), and sustainment for BACK-S or BACK-E. Quantitative predictors of sustainment include the CSAT score at a school-level across UH3 years 2–4, as well as contextual factors of each region. Contextual factors considered for this model will include urban versus rural location and school district size. We will evaluate these factors’ contribution to actual sustainment and to CSAT scores across implementation study arms.

In addition, we expect our qualitative and mixed methods analyses will identify how and why implementation strategies used for local uptake and sustainability vary in their impacts due to differences in contextual factors. Lessons learned will be incorporated into a BACK dissemination playbook co-developed with our community partners in a program sustainment phase, so that future communities can choose to implement BACK in a way that addresses local factors critical for success and sustainability.

### Implementation science framework

For this hybrid type 2 implementation-effectiveness trial, we are using PRISM to guide our approach and evaluation across our 4 study phases of Exploration, Preparation, Implementation and Sustainment [37–39, 43–46]. PRISM includes both the contextual determinants of successful implementation, as well as guidance on how to assess the Reach, Effectiveness, Adoption, Implementation and Maintenance/Sustainment (RE-AIM) outcomes with attention to health equity and representativeness [39, 43–45]. The PRISM contextual determinants are multi-level and include: the characteristics and perspectives on the intervention of inner setting school nurses/staff, implementers of ANavs, as well as children/families, the implementation and sustainability infrastructure, and the external environment [39, 43–45].The implementation and sustainability infrastructure for BACK includes local resources available to support initial implementation and sustained delivery of BACK. The external environment includes policies, regulations and incentives that support or hinder implementation and sustainment of BACK. Our Implementation Research Logic Model (see Fig. 2) outlines our key PRISM findings from our Exploration and Preparation phases on the left-hand side, the implementation strategy packages (BACK-S vs. BACK-E) that we will test, the core BACK intervention functions, expected mechanisms that were priority process measures identified by our CABs, and our RE-AIM outcomes that we expect to improve with the delivery of BACK. Further details on the BACK intervention and the BACK-S and BACK-E strategy packages are provided below.

### Study design

**Fig. 3:**
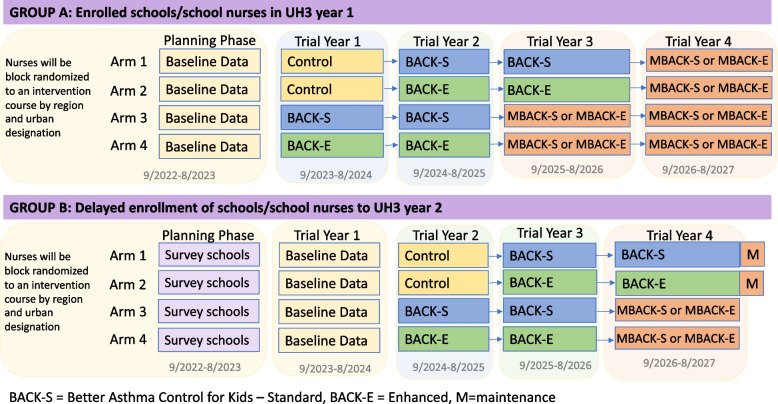
Study design for DECIPHeR Colorado program. In this diagram, there are two groupings of schools, those in GROUP A that had organizational readiness to implement either BACK-S or BACK-E in Year 1 of the funded trial, and those in GROUP B needed an additional year to prepare for implementation. The rows in each group represent the study arms for randomization. The columns are the study years, where the first column is the final year of the UG3-funded planning phase that included baseline data collection for the schools/school nurses in GROUP A, and Years 1–4 are the UH3-funded hybrid implementation-effectiveness trial period. BACK-S indicates the standard Better Asthma Control for Kids (BACK) package of implementation strategies of Tailor and adapt to context, Facilitation and Training, and BACK-E represents the enhanced strategy package of BACK-S strategies plus network weaving and consumer engagement. After 2 years of implementation, schools transition into the Maintenance phase where we will assess if they sustain either BACK-S or BACK-E, designated as MBACK-S or MBACK-E; for Arms 1 and 2 in Group B those are MBACK-S and MBACK-E, respectively

In this hybrid type 2 implementation-effectiveness trial, cluster randomization will occur in a parallel group approach, with a phased-in enrollment to BACK for all study arms [40, 47]. Figure 3 provides an overview of the study timeline and arms. In brief, children with uncontrolled asthma will be identified and recruited annually across the control phase and a two-year period of study team-supported implementation of either BACK-E or BACK-S. A comparison of the implementation strategies for BACK-E and BACK-S is provided in Table 2.

**Table 2:** Comparison of Implementation Strategies in BACK-S and BACK-E

DOMAINS	***BACK-Standard (BACK-S)*** ***implementation strategy package for program implementation phase***^**a**^	***BACK-Enhanced (BACK-E) implementation strategy package* for program implementation phase**^**a**^
DEFINITIONS	1. **Tailor and adapt** the Implementation Blueprint package of strategies – this Blueprint includes the **Facilitation** and **Training** strategies detailed here 2. **Facilitation**: ongoing learning communities for ANavs, nurses, and healthcare providers to support problem-solving 3. **Training**: asthma care management skills-building for ANavs and school nurses	BACK-Standard Strategy package (see middle column) + Enhanced strategy to engage consumers and promote network weaving at two socioecological levels: • Community level: **Promote network weaving** and engagement by navigator with community social determinants of health (SDOH) resource agencies through an existing statewide network of regional health connectors • Child/Family level: **Increase engagement** with child/family through school/family communication forums
ACTOR	1. **Tailor and adapt**: Research team with input from State and Community Advisory Boards 2. **Facilitation**: Research team 3. **Training**: Research team-directed, leveraging existing online training resources and the state health navigator program	BACK-Standard Strategy package (see middle column) + **Promote network weaving**: ANav + regional health connector **Increase engagement**: ANav
ACTION	1. **Tailor and adapt**: Blueprint for how to operationalize implementation strategies will be revised annually based on recommendations from navigators, school nurses, participating families/children and health care providers 2. **Facilitation**: Lead problem solving within learning collaboratives for specific roles (i.e., Asthma navigator Communities of Practice and Learning (COPL), School nurse COPL, and All-Hands COPL of navigators, nurses and health care providers) 3. **Training**: Provide ongoing training to improve asthma care management skills, including proper inhaler technique, and the use and interpretation of asthma control assessments/Asthma Intake Form	BACK-Standard Strategy package (see left column) – including a separate learning community for BACK-E Asthma navigators + **Promote network weaving**: ANav meets with a regional health connector in the existing statewide network for warm handoff introductions to six local community SDOH resource agencies **Increase engagement**: Asthma navigator submits six messages to a school-family forum (e.g., newsletter, school social media, or school event) to promote knowledge of local SDOH resources and accurate understanding of asthma
JUSTIFICATION	1. **Tailor and Adapt**: Informed by our EPIS and PRISM/RE-AIM implementation science frameworks 2. **Facilitation**: Critical for implementing our BACK program in past schools 3. **Training**: Critical for implementing our BACK program in past schools	Evidence from the literature [48, 49] and Community Advisory Board recommendations for this strategy to impact Reach and Equity

^a^Strategies in this table are for the Implementation phase (UH3 Trial Years 1–3) for schools served by school nurses randomized to either BACK-Standard or BACK-Enhanced

The implementation strategies for the planning phase (UG3 Planning phase years 2–3 and UH3 Trial Year 1) are common to both BACK-Standard and BACK-Enhanced and are not displayed here. For reference, those planning phase strategies were also part of the implementation blueprint developed in the planning phase and included training; engaging consumers; supporting clinician teams in schools and healthcare through development of communication templates and other resources stored in an online implementation guide; change infrastructure included development of online resource to support asthma case management by facilitating triage of asthma severity and supporting audit and feedback

### Intervention

The BACK intervention is a multiple level and multi-component intervention involving students and families with asthma, school nurses, community health care providers, and community agencies with resources to address SDOH. BACK is delivered by school nurses and ANavs according to the core intervention functions listed in Table 1. As in our prior school-based asthma programs, the intervention dose is three ANav visits for each student with asthma at their school and three ANav visits with their parent/guardian in-person, or by videoconference or telephone [18, 19]. ANav intervention activities include a standardized assessment to identify asthma education, asthma care and SDOH needs, and the resultant development of an individualized tailored plan of care. All BACK visits include asthma education reinforced with the provision of asthma educational materials; case management and care coordination to support successful asthma management at school and at home. The initial BACK visit includes assessment of SDOH needs related to asthma care (e.g., access to asthma care, transportation, and medications) [50], and ANavs provide community referrals to support any identified needs.

### Implementation strategy packages

Implementation strategies for the UH3 trial were developed collaboratively between the research team and CABs, by considering local PRISM contextual factors, priority outcomes of success and how best to deliver BACK to accomplish these priority outcomes. These strategies are delivered in one of two study arms as the BACK-S package or the BACK-E package. Both packages are delivered by an ANav in partnership with the school nurse-led team. The BACK-S package includes a tailor-and-adapt to context strategy of approaches identified as necessary to implement BACK in schools, including Facilitation and Training (see Table 2) [51–53]. BACK-S plans for implementation were operationalized as an implementation blueprint in the planning phase (2020–2023). Tailoring of this blueprint to the local context will occur through our Facilitation strategy that promotes adaptability of forms while maintaining fidelity to core functions of BACK-S and BACK-E, and an annual program evaluation process that may identify new strategies needed to tailor-and-adapt to context. Our Facilitation approach supports problem-solving through required weekly community of practice meetings for ANavs, optional learning collaborative meetings for school nurses (3 or more times yearly) and an “all hands meeting” annually of ANavs, school nurses and champions from local clinics – see “Researcher Team and implementer roles and responsibilities” for details on Facilitation leaders. The BACK-E package includes the BACK-S package plus an Enhanced Strategy package of consumer engagement with students/family through school-wide communications and network weaving to develop interrelationships to address SDOH (see Table 2). Our CABs voted to add these Enhanced strategies based on perceptions that increased school and community involvement were both feasible and likely to increase family willingness to participate.

### Research team and implementer roles and responsibilities

The BACK intervention is adopted at schools and by school nurses and their team members and implemented by ANavs and school nurse-led teams (see Fig. 1). In addition, community partners play a key role by engaging with and supporting schools and families.

#### Research team

The multidisciplinary research team consists of asthma specialists, implementation science experts, community-engaged research experts, health equity research experts, clinical trial specialists, school asthma program leaders, qualitative methodology expertise, public health representatives, biostatisticians, program evaluators, data collectors, ANavs, and research staff.

#### Community partners

Intersectoral CABs in each of our 4 Colorado regions are our primary community partners. CAB members include school nurses, health care providers, family members of a child with asthma, and/or community SDOH agency members. We also have a State Advisory Board with a liaison from each regional CAB and other state-level advisors from the Colorado Department of Public Health and Environment, Colorado Department of Education, and State Network of Colorado Ambulatory Practices and Partners network of primary care providers. Additionally, we partnered with a state-wide, non-profit, agency (Trailhead Institute) that serves as a link between community organizations, schools and local public health agencies across Colorado.

#### School nurses/team responsibilities

Each school nurse-led team consists of a school nurse and their “health aides” and delegates that support asthma care provision in schools. For the BACK program, the nurse or delegate introduces the ANav to the school community (other team members, teachers, staff, students/families), identify students with asthma, assist with completion and interpretation of the AIF [41] that identifies asthma control and thus eligibility, and partner with ANavs to obtain the Colorado Asthma Care Plan for Schools and inhalers at school.

#### ANav role/responsibilities

The ANav is the “glue” of the BACK intervention, and provides asthma education, case management and care coordination across families, school nurses/teams, health care teams and community partners to address families’ unmet social needs related to asthma care.

#### Training for school nurses and ANavs

ANavs are trained in data collection and data quality standards, asthma education, care coordination and case management, health navigation and mobilization through community engagement. ANavs implementing BACK-E are also trained on how to conduct the elements of the enhanced implementation strategies. School nurses are trained on the provision of quality asthma care in schools (identifying children with asthma, assessing asthma, Colorado Asthma Care Plan, inhaler use), working with ANavs to coordinate quality asthma care provision, and using academic platforms to support asthma care in schools.

#### Facilitators and trainers

Our program facilitators and trainers include research team investigators with expertise in asthma and asthma in schools (MG, LC), research assistants (RA, AB), and experienced ANavs who have delivered our school-based asthma management program in the Denver area schools for over a decade. See “Implementation Strategy Packages” above for details on the Facilitation meetings.

### Study settings, populations, and invested community partners

Given this intervention seeks to improve health disparities, BACK exerts influence across multiple socio-ecological levels. Thus, it is important to assess the impact of the study across those multiple levels of adopting elementary schools and their staff, students with asthma and their families, and ANav implementers. Students with asthma and their families will be assessed for the Reach and Effectiveness outcomes. Elementary schools and their staff will be assessed for the Adoption, Implementation and Maintenance outcomes. Our specific operationalized assessments of each RE-AIM outcome and the PRISM contextual assessments are described below in the “*Methods – Outcomes/Data Collection Procedures” section.*

### Recruitment

#### Regional recruitment

During the 3-year DECIPHeR-funded planning phase (2020–2023), the study team formed regional CABs. Through CAB discussions, eligibility criteria (see Table 3) were refined; regional CABs also supported identification of key districts serving under-resourced populations. Initially, five regions were approached for involvement and formed a CAB. Four of the five regions were able to develop school nurse and district support for the program. The Southwest Colorado region was particularly hard-hit by the COVID-19 pandemic in terms of school nurse turnover. Despite quarterly CAB meetings, we were unable to consistently engage with school nurses in that region, resulting in school recruitment for the DECIPHeR-funded UH3 phase trial coming from four of the five regions. We continue to engage the fifth region in our State Advisory Board meetings and follow-up CAB meetings to determine if they can be brought in during our BACK sustainment phase.

**Table 3:** Eligibility criteria, study roles, and RE-AIM outcomes according to school, school nurse, student/family and ANav

Level of assessment	Eligibility criteria	Roles in the study	RE-AIM Outcomes assessed
Elementary school setting	Met regional criteria^a^ and school-level social need criterion^b^, as well as in-person student attendance (necessary for BACK intervention)	Setting for implementation of BACK	Adoption (setting level); Maintenance (setting level)
School nurse	Employed by an eligible school district	Identify children with asthma for navigators to outreach	Adoption (staff level); Implementation (Fidelity, Adaptation, Cost); PRISM contextual factors
Student/family	Poor asthma control^c^ (current or past 12 months); Attends a school that adopted BACK intervention	Receive BACK intervention – 3 visits for children and 3 visits for family	Reach (student); Effectiveness (student); Maintenance (student level); PRISM contextual factors
ANav (Implementer)	N/A—hired to deliver BACK intervention, strong local community engagement, previous client facing experiences, and bilingual English–Spanish preferred	Primary implementer; Delivers BACK intervention visits and coordinates with school, student/family healthcare and community resources	Implementation (Fidelity, Adaptation, Cost); PRISM contextual factors

^a^Regional criteria: located within one of 4 priority mid-size urban and rural regions of Colorado identified in our UG3 planning phase. The priority for these regions was based on high community-level rates of unmet social needs, higher rates of free-and-reduced lunch and demographic representation of Black, Indigenous and People of Color (BIPOC) as compared to the state levels overall, no prior implementation of BACK, and Community Advisory Board (CAB) engagement with local school nurse champions to support adoption of BACK

^b^Social need criterion: met one of 2 criteria: 1) free-reduced lunch rates > 32% or 2) rural location based on Colorado Department of Education assessment during UG3 planning year 3 – these data will be reviewed annually during the UH3 trial to identify newly eligible schools in our 4 participating regions

^c^Poor asthma control as determined by Colorado Department of Education’s AIF. [41] Criteria for past poor asthma control in the last 12 months include one or more of the following: ≥ 2 urgent care/emergency department visits, ≥ 1 hospitalization; ≥ 2 courses of systemic corticosteroids; ≥ 5 school days missed due to asthma); Criteria for current poor asthma control include one or more of the following: reliever/rescue inhaler use > 2 days per week (not counting pre-treatment prior to vigorous activity), daytime asthma symptoms > 2 days per week, nighttime awakenings due to asthma > 2 nights per month, or activity limitation due to asthma “most of the time” or “all of the time

UH3 phase trial recruitment occurs across the levels detailed in Table 3, and outlined here.

#### School setting and school nurse recruitment

School districts meeting eligibility criteria (see Table 3) within our four regions were identified using the Colorado Department of Education school enrollment and characteristics database. Eligible school districts were invited to participate using email and phone. For those potentially interested in participation, a research team member (RA and/or LC) held meetings with school district officials and/or school nurses to provide additional information and discussion (e.g., flyer, e-mail, district-specific school nurse meetings). Regional CABs supported recruitment by advocating with school nurses and district officials.

#### Participant recruitment (students with asthma)

Recruitment processes for eligible students with asthma (see Table 3) were developed from prior processes [18, 19] and CAB input to support feasibility and program sustainability. These include: 1) BACK program intervention introduction to the school community via back-to-school nights or school registration attendance (ANav or research team member) and/or newsletters, 2) ANav 1:1 meeting with school nurses early in the school year, 3) Standard school nurse processes to identify children with asthma at registration or by review of their health database, 4) ANav outreach: contacting caregivers of children with asthma by phone call, texting, e-mail and/or postcard mailer to assess for student eligibility of uncontrolled asthma, and 5) ANav offering to meet with caregivers to discuss the BACK program.

#### Participant enrollment (students with asthma)

ANavs enroll interested and eligible students with uncontrolled asthma and their caregiver after discussing the BACK program and study, answering questions, and attaining informed consent from the caregiver and assent from the child. We propose enrollment of at least 300 students/families over the 4-year trial across the 4 regions. Study consent forms and enrollment processes were approved by the Colorado Multiple Institutional Review Board.

### Outcomes/data collection procedures

We will assess RE-AIM outcomes both quantitatively and qualitatively in a mixed-methods approach [40, 47]. We first describe our student level outcomes: the primary outcome of Reach (Table 4) followed by our Effectiveness outcome (see Table 5). Outcomes of adoption (see Table 6), implementation (see Table 7) and maintenance (i.e., school sustainment, see Table 8) include assessments at school setting, school staff and implementer levels. Analyses will compare the reach (primary outcome), student retention, adoption, costs to future adopters, and sustainment of schools between BACK-S vs. BACK-E overall and by region. Qualitative methods will identify contextual factors that predict student reach and retention, school-level adoption, costs to future adopters (schools), and sustainment for BACK-S or BACK-E.

**Table 4:** Reach

Outcome	Measure	Sample	Data collection procedures and Timing/Frequency
Primary Implementation outcome: Reach	% of eligible students enrolled The number of eligible students who consent are the numerator and the denominator is all eligible students from eligible schools	Eligible students	Numerator – annual numbers of enrolled students; Denominator – eligibility assessed annually in each school year by the Asthma Intake Form (see Table 3)
	Qualitative assessment of reasons for choosing to participate (or not)	Eligible students Families declining participation	Reasons for enrollment choices: annual interviews of caregivers; Reasons for not participating: annual asthma navigator tracking during recruitment
Representativeness of Reach (Reach- Equity)	Characteristics of sex, race, ethnicity, free and reduced lunch, and primary language of students who do participate as compared to school-wide demographics	Enrolled students School-wide demographics	Quantitative demographics from the annual baseline survey of consented participants; Quantitative demographics from annual reports to the Colorado Department of Education by participating school settings

**Table 5:** Effectiveness

Outcome	Measure	Sample	Data collection procedures and Timing/Frequency
**Primary health outcome:** Asthma exacerbations	# of exacerbations per year. Asthma exacerbation defined as any of the following for asthma: use of systemic steroid burst therapy, ED visit, or hospitalization	Enrolled students in control, as well as BACK-S and BACK-E intervention arms	Annual family surveys – baseline survey collected by ANav; 12-month follow-up survey captured by a data collector masked to study arm assignment [41]
School absence	Days absent due to asthma	Enrolled students in control, BACK-S and BACK-E	Annual family surveys – baseline survey collected by ANav; 12-month follow-up survey captured by a data collector masked to study arm assignment
Inhaler Technique	Accurate inhaler technique (1–5 scale)	Enrolled students in BACK-S and BACK-E	Captured at each student visit by direct observation of student technique by ANav
Perceptions of BACK program impact on student’s asthma health	Survey items assessing impact of BACK program on asthma control and asthma self-management	Enrolled students in BACK-S and BACK-E	Annual family survey – captured annually by masked data collector Annual interviews of a purposive sample of families

**Table 6:** Adoption

Outcome	Measure	Sample	Data collection procedures and Timing/Frequency
Setting level adoption^a^	% of eligible schools^a^ agreeing to participate; Adoption = schools randomized divided by all eligible schools	Numerator: Schools^a^ that agree to participate Denominator: Eligible schools	Numerator of Adoption (setting)^a^: all schools randomized to study arm assignment in a given school year Denominator of Adoption (setting)^a^: all eligible schools in a given school year—see Table 3 for eligibility criteria
	Representativeness of school settings (School demographic data include %free-reduced lunch, % English as a second language, race/ethnicity, # students enrolled, # school teachers, # school nurses/team members and school nurse team to student ratio)		Annual collection of data from Colorado Department of Education for schools that adopted as compared to eligible schools that declined adoption
	Qualitative reasons for participation (or not) by school or school district		Activity tracking by research team and ANavs re: reasons for participation or not
Staff level adoption	% of nurses from eligible schools (see above) agreeing to support the intervention	All eligible school nurses	Numerator of Adoption (staff): among school nurses whose school administration agreed to participate, the number of nurses who agreed (or were assigned) to be randomized to study arm assignment in a given school year Denominator of Adoption (staff)^a^: nurses whose school administration agreed to participate in a given school year—see Table 3 for eligibility criteria
	Representativeness of school nurses (education, years of service, age-range, race/ethnicity, number of schools served)	Participating nurses compared to representative sample of statewide school nurses	Participating nurses: Annual school nurse survey (enrolled) Aggregate demographic data of Colorado school nurses
	Feasibility, acceptability, and appropriateness [54]	Enrolled School Nurses	Annual surveys from all enrolled school nurses Feasibility, acceptability and appropriateness will be further explored in annual qualitative interviews with a purposive sample of school nurses

^a^Note: if a school district made decisions rather than an individual school administrator, these data will instead be reported at the level of the school district setting

**Table 7:** Implementation

Outcome	Measure	Sample	Data collection procedures and Timing/Frequency
Fidelity and Completeness to the intervention	% of participants receiving adequate dose of the intervention (Retention for at least 2 of 3 planned visits for both children and caregivers)	ANav Intervention visits with enrolled Children and Caregivers	ANav Intervention Task Tracking (ongoing)
Quality of intervention delivery	Rubric of quality level for each intervention function (high, acceptable, below, unacceptable) Qualitative assessment of patient-centered communication by intervention visit recordings Family Perspectives on intervention delivery quality	School nurse (identification of students with asthma for ANav) and ANav (other functions) ANav intervention visits with caregivers Families	Research team assessment based on ANav data captured for each intervention function Zoom/audio-recordings of intervention visits reviewed by research team qualitative data analyst Annual family interviews
	Family satisfaction with core intervention functions and net promotor score for the BACK program [55]		Annual family program evaluation survey
Fidelity and Completeness of the Implementation Strategy	% implementation strategies completed during each school year as defined in the implementation blueprint, with a focus on Training, Facilitation, and the Enhanced implementation strategies	ANavs	Annual assessment of the rate of implementation strategy completion for that school year: informed by ANav task tracking and training and meeting attendance, and agenda and minutes from meetings (e.g. communities of learning and practice, CAB, SAB, annual research mtg, periodic reflections)
Quality of implementation strategies	Perspectives of nurse, family and ANavs on implementation strategy success as defined by enhanced communication and engagement, care coordination, sufficient training, and tailor and adapting to context as appropriate	School Nurse and Family ANav	Annual interviews of purposive sample: school nurse, family caregiver Annual interviews: all ANavs
	Quantitative assessment of overall program satisfaction and satisfaction with program implementation resources	Family, School nurse, ANav	Annual program evaluation survey
Adaptations to intervention and implementation strategies	Modifications to design or delivery of BACK core functions for intervention, implementation strategies, or study design	ANav	Ongoing assessments at weekly ANav COPLs and specifically assessed during quarterly periodic reflections by ANav using a research team tracking tool
Cost	Resources used	Supplies used for BACK	Invoices
	Cost outputs—School staff time and ANav time	School staff involved in BACK implementation, ANav	Ongoing ANav activity tracking, annual Navigator and school nurse surveys, invoices, attendance at school Nurse and Navigator COPLs
	Cost inputs – reimbursement	School district administrators	Study team annual tracking

**Table 8:** Maintenance

Outcome	Measure	Sample	Data collection procedures and Timing/Frequency
Maintenance—Schools that continue to offer the BACK intervention	School nurses continue to deliver the BACK intervention (either BACK-S or BACK-E)	Randomized schools	Annual program continuity tracking
Sustainability	Clinical Sustainability Assessment Tool (CSAT)	Enrolled school nurses, ANavs	Annual survey with ANavs, school nurses and healthcare provider liaisons during an all-hands community of practice and learning (COPL)
	Qualitative focus group and interviews	Enrolled school nurses, ANavs, and healthcare provider champions	Annual interviews will be conducted with all ANavs and a purposive sample of school nurses. At the end of the school year, an all hands COPL, will elicit sustainability discussions with healthcare providers, ANavs and school nurses

This section provides an overview of data collection methods and procedures, including tracking of outcomes and tasks, surveys and interviews. Further details are provided for each outcome assessment below.

- Task tracking will be ongoing and completed by ANavs in both BACK-S and BACK-E arms; tracking of relevant items in the usual care arm are completed by masked data collectors.
  - Tracked outcomes include reasons for students/families participating (or not), intervention activities, time spent in each visit with the student and family, and implementation-related activities.
- Annual surveys will be sent in the second half of each school year to ANavs, school nurses, families, and health care providers. Surveys will be sent by e-mail from a REDCap online database with up to 2 follow-up contacts for completion over a 2-month period.
  - Survey items are tailored to the target population and outcome assessed and are defined in more detail for each RE-AIM outcome in Tables 5, 6, 7 and 8.
- Enrolled caregivers of students with asthma will complete the following assessments:
  - Baseline assessment that includes:
    - Identical questions from the AIF asked to confirm eligibility
    - Health outcomes at the time of program enrollment
    - Current asthma care, knowledge, self-management and barriers/facilitators to care
    - Unmet SDOH needs that may influence asthma management
-
  - Annual end-of-school year program evaluation survey
  - Annual assessment of health outcomes completed by a data collector masked to study group, with support of translators for non-English speaking caregivers, if warranted [41]
- Annual interviews will be conducted with all ANavs, and a purposive sample of school nurses, and students/families with higher and lower recommendations to use BACK (i.e., Net Promoter score) [55].
  - Interviews will explore topics of acceptability, appropriateness, and perceptions of the program, experiences with program reach, intervention quality, effectiveness, and reasons to continue with the program in the future or not.

### Primary outcome: reach (student level)

Reach is defined as the proportion and representativeness of eligible students who enroll in BACK. Reach will be assessed as a dichotomous variable identified as yes for those who consent, and no for those who do not consent during the enrollment period. ANavs will track reported reasons families are willing/not willing to participate. In terms of retention, ANavs will track reasons for participant dropout and will offer a brief interview to examine these reasons further.

#### Secondary outcome: effectiveness (student level)

Effectiveness is defined as the impact of the BACK program on asthma health in students (see Table 5). The health outcome of greatest interest to our BACK community partners (and in our prior studies) is asthma control, operationalized as annual exacerbation rates (primary health/effectiveness outcome). Additionally, we will evaluate a secondary health/effectiveness outcome of school absences due to asthma. We will compare our effectiveness outcomes among students with uncontrolled asthma randomized to either usual care (control) or BACK-S or BACK-E. Additionally, family perception of effectiveness will be obtained from families receiving active BACK intervention through annual survey assessment and further explored with families completing interviews.

#### Secondary outcome: adoption (setting level)

Adoption is defined as the proportion and representativeness of settings and staff who work in these settings, respectively, that agree to deliver the intervention [43, 45]. Adoption measures are described in detail in Table 6, and these include both quantitative and qualitative data. As noted above in Table 3, we define our adoption setting at the school level and our staff who adopt BACK at the school nurse level – the nurse role is critical to identify students with asthma, and they partner with ANavs who deliver other BACK intervention functions. Additionally, we will examine Adoption-related factors through annual survey measures and qualitative interviews: feasibility, acceptability and appropriateness. In terms of representativeness, Colorado Department of Education data and school characteristics will be used to identify representativeness at the school setting level — see Table 6 for examples.

#### Secondary outcome: implementation (school setting staff and ANav level)

Implementation is defined as fidelity to the intervention’s core functions (see Table 1) in consistency and quality, structured assessment of any adaptations made according to standard criteria, and the time and costs of the program [56, 57]. The implementation measures and data collection procedures are described in Table 7 below. Fidelity completeness and quality will be assessed for intervention core functions and both implementation strategy packages. We will use a mixed methods approach to examine fidelity; family and school nurse input in annual interviews will provide key insights.

To assess Adaptations, BACK Facilitators will complete short debrief summaries following each Facilitation discussion; these will be mined for emergent topics related to intervention and implementation adaptations. Adaptations will be discussed bi-weekly by study team members, and tracked based on standard implementation methods [56–59]. Adaptations will be reviewed with the full study team annually (with appropriate masking) to inform tailoring the program to context in the upcoming year while preserving the core functions of the intervention and avoiding adding any BACK-E implementation strategies to the BACK-S package.

To measure program costs, activities will be defined by developing a process map and quantified through ongoing tracking of intervention and implementation activities, attendance, annual surveys, and time estimates, per standard time-based activity costing methods [60]. We will work with ANavs and school nurses annually to track both BACK program costs and any reimbursements/incentives received by schools for BACK visits, such as Medicaid reimbursements.

#### Secondary outcome: maintenance (setting level)

Maintenance is defined as the extent to which a program becomes accepted practice within the setting, and this is operationalized as a school continuing the BACK intervention (either BACK-S or BACK-E) after 2 years of active implementation support. Maintenance will be captured using a mixed methods approach as described in Table 8. For qualitative assessments, a structured focus group guide will be used, and sessions will be audio recorded, transcribed, and analyzed. The research team, school nurses, ANavs, and health care providers will complete the validated clinical sustainability assessment tool annually [61]. With appropriate masking, CSAT and qualitative results will be discussed by the study team at the dedicated annual program evaluation meeting and explored with CABs to further pursue opportunities to enhance program sustainability. School sustainment of any BACK core functions (see Table 1) during the maintenance phase will be tracked annually by a data collector masked to study arm assignment — with attribution of who delivers each BACK function (e.g., ANav, school nurse).

### Methods – analysis and power calculation for aims 1 and 2

Our type 2 hybrid trial will address our overarching hypotheses and research questions regarding comparative implementation outcomes to inform future schools’ decisions to adopt or sustain BACK, and the effectiveness of BACK on asthma control across a set of rural and urban schools [47, 62]. By phasing in the active BACK implementation packages the study contains a control group for one year. This allows a comparison of both BACK-S and BACK-E to usual asthma care, a comparison of great interest to our CAB members, school communities and investigators.

The phased-in, parallel group randomized trials (GRTs) include both a 3-arm trial and a 2-arm trial. The primary aim of the 3-arm trial is to compare the health outcomes of each arm to control (no BACK). Implementation outcomes cannot be compared in the 3-arm trial, as the primary implementation measure of reach will not be evaluated in the control arm. The primary aim of the 2-arm trial is to compare implementation outcomes and health outcomes between the two arms of BACK-E and BACK-S (Fig. 3). We refer to these trials as the ‘3-arm trial’ and the ‘2-arm trial’ throughout the analysis section.

The primary implementation outcome is reach (see Table 5). The primary health outcome is number of asthma exacerbations in the previous year (see Table 6).

For statistical analysis of Aims 1 and 2, the primary implementation aim and primary health outcome aim of interest can be written as the following null hypotheses for modeling and testing either the 3-arm trial or the 2-arm trial:

A. In a 3-arm trial, after one year of implementing the programs the null hypotheses for the health outcome of interest are:
  1. no difference between the incidence rates of asthma exacerbation for BACK-S compared to controls.
  2. no difference between the incidence rates of asthma exacerbation for BACK-E compared to controls.
B. In the 2-arm trial, after one year of implementing the programs, the null hypotheses for the primary implementation outcome of interest and the primary health outcome of interest between implementation arms are:
  1. no difference in the odds of reach between BACK-S compared to BACK-E. (Primary hypothesis of interest)
  2. no difference in the rates of asthma exacerbation between BACK-S compared to BACK-E (Secondary hypothesis; Primary health outcome is rates of asthma exacerbations)

The null hypotheses will be assessed using a generalized linear mixed model [63, 64]. The primary implementation outcome is binary and the primary health outcome is a count. Analytic models of the implementation outcomes will be longitudinal mixed models. Analytic models of health outcomes will be constrained longitudinal data analysis models where baseline measures are included in the vector of outcomes. [65] Models will allow for time varying effects and correlation via nested random coefficient effects, and will include time-varying random effects for students, school, and ANavs in any model for continuous or count outcomes. The regression model will adjust for member level covariates of age, sex and insurance provider.

Power and sample size estimates were calculated using the GRT Sample Size Calculator available at the NIH website [66]. For both comparisons of interest, we fixed power at 90% and focused on the simple difference in rates. The power analysis also assumed an ICC of 0.05, based on three years of data from our Denver Metropolitan Area program in 6 school districts. Expected rates of asthma (9%), AIF [41] completion (64%), eligibility (~ 15 children per nurse) and engagement also come from the Denver Metropolitan Area program numbers [20].

For Aim 1, the 2-arm trial of the primary implementation outcome of reach rate (anticipated lower bound on reach rates of 33%), a power and sample size analysis of a parallel GRT indicates that 30 nurse clusters in each arm with 5 individuals per cluster is powered at 90% to detect differences in the reach rates of at least 20% for a Type 1 error rate of 0.05.

For the Aim 2, the 3-arm trial of the health outcome of asthma exacerbation (expected baseline rate of 1.5 exacerbations per student per year for usual care), a power and sample size analysis of a parallel GRT indicates that 20 clusters (school nurses) in each arm with 5 individuals per cluster is powered at 90% to detect differences in asthma exacerbation rates of at least 0.75 exacerbations per person year (50% reduction) at the adjusted Type I error rate of 0.025 for two comparisons.

### Analysis of secondary outcomes

Evaluation of the RE-AIM outcomes, community engagement outcomes, and health-related outcomes will involve the collection and analysis of both quantitative and qualitative data. These data will be analyzed and integrated according to our research group’s published methods for qualitative assessments of RE-AIM outcomes, including specific methods to measure adaptations, fidelity, and implementation costs [67–70]. Measures for each RE-AIM outcome are described in detail in Tables 4, 5, 6, 7 and 8 above. Community engagement outcomes include the number of health care partner organizations to support asthma care and the number of SDOH partner organizations and the strength of partner relationships [71] for each navigator. Methods for quantitative and qualitative data collection and our mixed methods approach for secondary outcomes are described below.

### Setting level adoption, implementation and maintenance analyses

#### Quantitative data analysis for aims 1 and 2

Analysis will compare adoption rates by settings and staff who do and do not agree to participate using t-tests, Fishers exact test, and Wilcoxon rank sum as appropriate for each measure (i.e., representativeness). Fidelity rates will be assessed using a percentage of completeness of at least 2 visits in all eligible students [72]. Survey measures described in Tables 5, 6, 7 and 8 will be analyzed using methods established for each validated measure (e.g. CSAT [61]) and descriptive statistics including percentages, means, and standard deviations for novel measures (e.g. patient satisfaction) [54, 55, 61].

We will evaluate intervention and implementation costs (see Table 7) using a time driven activity-based costing approach (TDABC) from the payer perspective [60, 73]. As recommended by StaRI we will separately assess costs for the BACK intervention and for the implementation strategies, including explicit assessment of the additional costs of the enhanced implementation strategy [74]. For community engagement, we will compare the number of partnering SDOH and asthma care (primary care provider and specialty clinics) organizations and the strength of relationship at baseline (UG3 year 3) and in subsequent UH3 trial years.

#### Quantitative data analysis for aim 3

Our third aim will identify PRISM contextual factors [39, 43] from qualitative data (see Fig. 2) that predict student reach and retention, school-level adoption, costs to future adopters (schools), and sustainment for BACK-S or BACK-E. Regression models will assess the effects of PRISM contextual level factors upon each implementation outcome, including school characteristics (e.g., school district size). Data will be summarized, and models will assess differences in expected implementation outcomes by cross-sectional levels of contextual factors. Quantitative predictors of sustainment will also include repeated annual measures of CSAT [61]. We will evaluate the contextual factors’ contribution to each implementation outcome by implementation study arm.

#### Qualitative data analysis for aims 1–3

We will use a modified grounded theory methodology and will employ a combination of inductive and deductive approaches to coding and analysis. Interviews will be audio-recorded, transcribed verbatim, and managed with ATLAS.ti23. We will use a team-based approach to coding and analysis. We will follow best practices for virtual semi-structured interviews and thematic content analysis techniques [75–77]. The qualitative team will meet regularly throughout coding and analysis to develop shared interpretations of the data, reveal and check biases and assumptions, develop themes, and finalize results. We will present preliminary findings to CABs and other stakeholders to include their interpretation before finalizing findings [78].

#### Mixed methods approach and analysis

**Fig. 4:**
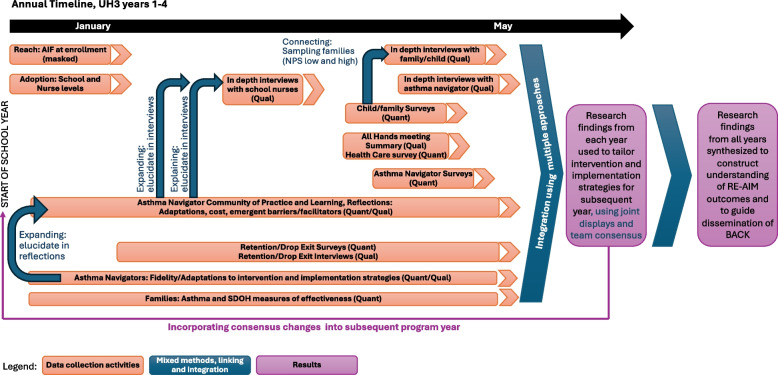
Annual Timeline for Mixed Methods data collection and analysis. This figure depicts the timing of qualitative and quantitative data collection and analysis over the course of each study year, and the planned use of these data. Abbreviations: AIF (Asthma Intake Form), Qual (qualitative), Quant (quantitative), BACK (Better Asthma Control for Kids intervention), NPS (net promoter score – level of recommendation of BACK), SDOH (social determinants of health)

Using a complex convergent mixed methods design, each element of data collection will typically occur separately, meaning the quantitative data will be collected and analyzed and the qualitative data will be collected and analyzed [79, 80]. Then the two sets of data will be analyzed and interpreted together by using a matrix approach to mixing the data and primary analysis integration strategies of expanding, explaining and connecting as shown in Fig. 4 and joint displays will be created [81–84].

The end result of this mixed-method analysis will be to identify contextual factors that predict RE-AIM outcomes, and to follow approaches recommended by Shelton, Chambers and Glasgow to identify health equity considerations for schools randomized to BACK-E compared to BACK-S [85].

### Methods—development of playbook to support sustainment

With CAB input, we already developed an online implementation guide that will be adapted to support sustainment and future dissemination of BACK based on this trial’s findings. We will refine the resultant dissemination playbook to describe the relative impact and cost of using BACK-S or BACK-E for a given context. Briefly, this guide will be based on the findings on our RE-AIM mixed methods analysis of how specific contextual typologies of communities (e.g., community size (urban, suburban, rural) influenced our results. Overall, this playbook will inform decisions about whether to adopt BACK for different typologies of school districts, schools, students and families and communities.

## Discussion

This study has potential to impact both pediatric asthma disparities and the field of IS. For the field, by comparing our implementation outcomes between BACK-E vs. BACK-S, we will determine if the addition of the enhanced strategy package (BACK-E) to promote further school/community engagement yields additional benefits in reach, retention and other implementation outcomes. Regarding asthma disparities, we expect the BACK program will address inequities by improving asthma control and associated morbidity, and we will test this hypothesis using a control group. Taken together, these data will inform future communities and schools to decide whether or not the benefits of BACK are worth their investment.

## Conclusion

This community-engaged trial will test the impact of BACK to reduce pediatric asthma disparities. It will also develop key products to disseminate BACK more broadly, including a dissemination playbook to accelerate sustainable dissemination of BACK to other communities experiencing health inequities in childhood asthma.

### Supplementary Information

Supplementary Material 1.

Supplementary Material 2.

## Data Availability

Not applicable as there are no data in this protocol manuscript.

## Abbreviations

SDOH: Social Determinants of Health
BACK: Better Asthma Control for Kids
RN: School Registered Nurse
ANav: Asthma Navigator
ED: Emergency Department
RCT: Randomized Controlled Trial
EPIS: Exploration, Preparation, Implementation and Sustainment Model
DECIPHER: Disparities Elimination through Coordinated Interventions to Prevent and Control Heart and Lung Disease Risk
PRISM: Pragmatic Robust Implementation Sustainability Model
CAB: Community Advisory Board
BACK-S: BACK-Standard
BACK-E: BACK-Enhanced
RE-AIM: Reach, Effectiveness, Adoption, Implementation, and Maintenance
AIF: Asthma Intake Form
