# Supplementary material for: Reducing asthma attacks in disadvantaged school children with asthma: study protocol for a type 2 hybrid implementation-effectiveness trial (Better Asthma Control for Kids, BACK)
Source: Implement Sci. 2024 Aug 15;19:60. doi: 10.1186/s13012-024-01387-3 (PMC11325631; doi:10.1186/s13012-024-01387-3)
Supplement: Supplementary file 1 — Supplementary Material 1. [file 13012_2024_1387_MOESM1_ESM.docx]

Supplemental Figure 1. **Expanded CONSORT for the Better Asthma Control for Kids (BACK) Trial**

**Contextual Considerations**

**Content**

**Selected RE-AIM Dimensions**

Schools in Selected Regions (n)

Colorado Settings (schools) Eligible (n)

Colorado Settings (schools) excluded due to not meeting at least 1 inclusion criteria (n)

Schools^#^ that decline

(n, %, reasons)

Adoption: Nurse clusters (n, %) serving schools (n, %) randomized to one of 4 BACK study arms

(n and %)

Schools^#^ interested in participating (n)

schools) Eligible (n=)

Students with asthma **identified** (**n** from N schools)

Delayed BACK-E (n) schools served by randomized Nurse clusters (n)

BACK-E (n) schools served by randomized Nurse clusters (n)

Delayed BACK-S (n) schools

served by randomized Nurse clusters (n)

**Eligible** students with uncontrolled asthma **(n)**

**Eligible** students with uncontrolled asthma **(n)**

**Eligible** students with uncontrolled asthma **(n)**

**Eligible** students with uncontrolled asthma **(n)**

**CONSENTED AND ENROLLED STUDENTS (n)**

BACK-S (n and %)

Delayed BACK-S (n and %)

BACK-E (n and %)

Delayed BACK-E (n and %)

**FOLLOW-UP (n)**

BACK-S (n and %)

Delayed BACK-S (n and %)

BACK-E (n and %)

Delayed BACK-E (n and %)

**ANALYZED STUDENTS (n)**

BACK-S (n and %)

Delayed BACK-S (n and %)

BACK-E (n and %)

Delayed BACK-E (n and %)

Schools where BACK was continued/modified after 2 years of implementation support

(n, % and reasons)

Schools where BACK was discontinued

(n, % and reasons)

***Adoption Representativeness***

*Setting level:*

*School characteristics of adopters vs non-adopters*

*Staff level:*

*Characteristics of school nurse adopters vs non-adopters*

***Reach Representativeness***

*Differences between demographics of students enrolled compared to eligible schools, and between conditions*

*Reasons any students were eligible but not consented or enrolled (n=); any differences by study arm*

*Reasons any students did not have data available to analyze (lost to follow-up: n=)*

*Reasons any students were not included in the analysis (n=)*

*Characteristics of schools who continue vs not continue*

Schools could not meet participation requirements

(e.g., lacked infrastructure for Asthma Navigator to access school ): (n, % reasons)

**ADOPTION**

Schools^#^ agreed to participate (n)

schools) Eligible (n=)

Staff Level: School nurses that decline

(n, %, reasons)

BACK-S (n) schools served by randomized Nurse clusters (n)

Students with asthma **identified (n** from N schools)

Students with asthma **identified** (**n** from N schools)

Students with asthma **identified** (**n** from N schools)

**REACH**

**REACH AND EFFECTIVENESS**

**MAINTENANCE**

Supplemental Figure 1 Legend: This Expanded CONSORT diagram was adapted from Glasgow RE, Huebschmann AG, Brownson RC. Expanding the CONSORT Figure: Increasing Transparency in Reporting on External Validity. *Am J Prev Med*, 2018 (PMID: 30033029).

# Note: if a school district made decisions rather than an individual school administrator, these data will instead be reported at the level of the school district setting

*Summary: Reach and other implementation outcomes are compared between the two study groups of BACK-Standard (BACK-S) and BACK-Enhanced (BACK-E) (i.e., 2-arm trial: for details, see “***Methods – Analysis and Power Calculation for Aims 1 and 2***) . The delay of program implementation for a subset of schools creates a control group that will allow a comparison of effectiveness outcomes between control, BACK-E and BACK-S study arms after one year (i.e., 3-arm trial: for details, see “***Methods – Analysis and Power Calculation for Aims 1 and 2***). The student control data are collected in the first year of enrollment in either the Delayed BACK-S or Delayed BACK-E study arm.
